# High-Throughput Assay of Cytochrome P450-Dependent Drug Demethylation Reactions and Its Use to Re-Evaluate the Pathways of Ketamine Metabolism

**DOI:** 10.3390/biology12081055

**Published:** 2023-07-27

**Authors:** Nadezhda Y. Davydova, David A. Hutner, Kari A. Gaither, Dilip Kumar Singh, Bhagwat Prasad, Dmitri R. Davydov

**Affiliations:** 1 Department of Chemistry, Washington State University, Pullman, WA 99164, USA; n.davydova@wsu.edu (N.Y.D.); david.hutner@wsu.edu (D.A.H.); 2 Department of Pharmaceutical Sciences, Washington State University, Spokane, WA 99202, USA; kari.gaither@wsu.edu (K.A.G.); dilip.singh@wsu.edu (D.K.S.); bhagwat.prasad@wsu.edu (B.P.)

**Keywords:** cytochrome P450, formaldehyde, demethylation, high-throughput assay, human liver microsomes, ketamine, CYP2C19, CYP3A, CYP2D6, CYP2B6

## Abstract

**Simple Summary:**

Here, we introduce a reliable, inexpensive, and versatile method for high-throughput kinetic assays of drug metabolism based on fluorometric quantification of formaldehyde (FA) formed in cytochrome P450-dependent demethylation reactions. We describe the implementation of this technique for automatized assays of cytochrome P450-dependent drug metabolism in human liver microsomes. We also report the use of our new approach for re-evaluating the pathways of metabolism of the NMDA-receptor antagonist ketamine, which is increasingly used as an antidepressant in the treatment of alcohol withdrawal syndrome. Probing the kinetic parameters of ketamine demethylation by 10 major cytochrome P450 (CYP) enzymes, we demonstrate that in addition to CYP2B6 and CYP3A enzymes, which were initially recognized as the primary metabolizers of ketamine, an important role is also played by CYP2C19 and CYP2D6. At the same time, the involvement of CYP2C9 suggested in the previous reports is insignificant.

**Abstract:**

In a search for a reliable, inexpensive, and versatile technique for high-throughput kinetic assays of drug metabolism, we elected to rehire an old-school approach based on the determination of formaldehyde (FA) formed in cytochrome P450-dependent demethylation reactions. After evaluating several fluorometric techniques for FA detection, we chose the method based on the Hantzsch reaction with acetoacetanilide as the most sensitive, robust, and adaptable to high-throughput implementation. Here we provide a detailed protocol for using our new technique for automatized assays of cytochrome P450-dependent drug demethylations and discuss its applicability for high-throughput scanning of drug metabolism pathways in the human liver. To probe our method further, we applied it to re-evaluating the pathways of metabolism of ketamine, a dissociative anesthetic and potent antidepressant increasingly used in the treatment of alcohol withdrawal syndrome. Probing the kinetic parameters of ketamine demethylation by ten major cytochrome P450 (CYP) enzymes, we demonstrate that in addition to CYP2B6 and CYP3A enzymes, which were initially recognized as the primary metabolizers of ketamine, an important role is also played by CYP2C19 and CYP2D6. At the same time, the involvement of CYP2C9 suggested in the previous reports was deemed insignificant.

## 1. Introduction

Assays of the activity of cytochromes P450 (P450s) in drug-metabolizing reactions are widely employed in pharmacological research. They constitute an ultimate part of the studies with newly developed drugs necessary for their approval for practical use [1]. Determining the rate of P450-dependent reactions is among the most frequently performed enzyme assays in current biochemical practice. The most common methods for these studies are based on detecting the products of P450-dependent reactions with the LC-MS/MS technique. They are often used in a high-throughput setup necessary for scanning large numbers of drug candidates. However, LC-MS/MS methods are time-consuming and laborious and require specific setups for each particular product being monitored. Therefore, developing a more universal and less resource-consuming method for high-throughput P450 activity assays would be highly beneficial. Our motivation for developing such a method is caused by our interest in high-throughput studies of correlations between the profile of drug metabolism and the composition of the P450 pool in the human liver. These studies are necessary for elaborating the concept of functional integration in the drug-metabolizing ensemble [2].

In our search for a reliable, inexpensive, and versatile technique for high-throughput kinetic assays of drug metabolism, we turned our attention to an old-school approach based on the determination of formaldehyde (FA), which is formed in the oxidative demethylation of drugs [3,4]. N- and O-demethylations constitute up to one-third of all reactions catalyzed by human P450 enzymes (see [5] for examples). The most common approach for monitoring the P450-dependent formation of FA is a colorimetric technique based on the Nash method [6] that uses the formation of diacetyldihydrolutidine, a heterocyclic compound absorbing in the blue-to-near-UV region, from formaldehyde, ammonium, and acetylacetone through the so-called Hantzsch reaction. Following the first publications of its use [7,8], this method became predominant in the early studies of P450 catalysis. However, due to its relatively low sensitivity, this technique was later superseded by HPLC- and LC-MS/MS-based techniques. 

Nevertheless, the capabilities of methods based on formaldehyde detection are nowhere near exhausted. There are a series of new sensitive techniques for colorimetric and fluorometric detection of FA (see [9,10] for a review), which may be used for monitoring P450-dependent reactions. We probed several of these techniques in this study and chose a fluorometric approach based on the Hantzsch reaction with acetoacetanilide. Here we describe a sensitive and robust high-throughput method for monitoring P450-dependent demethylation reactions using a liquid handling robot and fluorometric plate reader.

We also report the application of our new technique to re-evaluate the pathways of metabolism of ketamine by the cytochrome P450 ensemble of the human liver. This drug is an NMDA receptor antagonist widely used as a dissociative anesthetic and antidepressant. Our interest in the metabolism of ketamine, a typical example of a substrate of P450-dependent demethylation, is inspired by the fact that this drug is increasingly used as an antidepressant for the treatment of alcohol withdrawal syndrome (AWS) and alcohol use disorder (AUD) [11,12]. However, due to the narrow therapeutic index of ketamine used as an antidepressant, a better understanding of its metabolic pathways in alcohol-exposed patients is needed to expand its use further [13].

The only FDA-approved ketamine-based medication for treating AWS and other depressive disorders is Esketamine, a nasal spray containing S-ketamine, one of the two enantiomers of the drug [14]. Therefore, we elected S-ketamine as a substrate of P450-dependent demethylation reactions in this study. We studied its metabolism by ten recombinant human drug-metabolizing P450s. Our results make significant adjustments to our understanding of ketamine metabolism pathways. In addition to confirming the role of CYP3A and CYP2B6 enzymes, they indicate a significant involvement of CYP2D6 and CYP2C19, which was initially underestimated.

We also studied ketamine demethylation by five different pooled preparations of human liver microsomes (HLM), including a preparation from ten chronic alcohol consumers. We observed dramatic differences in the kinetic parameters of this reaction across the HLM samples. Notably, the activity of the pooled preparation obtained from alcohol-exposed donors was the highest of all HLM samples probed, and the shape of its substrate saturation profile (SSP) suggests an important difference from other HLM samples in the involvement of the individual P450 species.

In summary, our study introduces an efficient, robust, and inexpensive platform for high-throughput studies of drug metabolism by HLM samples, which is necessary for studying functional integration in the P450 ensemble. It also provides important new information on the pathways of metabolism of ketamine, a high-potential antidepressant for use in AWS and AUD.

## 2. Materials and Methods

### 2.1. Chemicals

Actoacetanilide was the product of the Tokyo Chemical Industry (Tokyo, Japan). S-Ketamine was obtained from Cayman Chemical Company (Ann Arbor, MI, USA) as a certified reference material (CRM) solution in methanol (1 mg/mL). Before using this reagent, the methanol solvent was evaporated under a stream of argon gas, and the chemical was redissolved in 0.1 M Hepes buffer pH 7.4 containing 60 mM KCl. The concentration of ketamine in the stock solution was determined from its absorbance at 265 nm using the extinction coefficient of 0.562 mM^−1^cm^−1^. (S)-norketamine hydrochloride and midazolam were purchased from Cayman Chemical Company (Ann Arbor, MI, USA). Glucose-6-phosphate dehydrogenase from *Leuconostoc mesenteroides*, NADP, Glucose-6-phosphate, and midazolam were the products of MilliporeSigma (Burlington, MA, USA). All other reagents were of ACS grade and used without additional purification.

### 2.2. Microsomes Containing Recombinant Human Cytochromes P450

Most microsomal preparations containing individual P450 enzymes were Supersomes^®^, the products of BD Gentest, formerly a part of Corning Life Sciences (Tewksbury, MA, USA). In the present study, we used preparations containing CYP1A2, CYP2A6, CYP2B6, CYP2C9, CYP2C19, CYP2E1, CYP3A4, and CYP3A5. All those preparations contained human CPR and cytochrome b_5_ co-expressed. The preparation of insect cell microsomes containing human CYP2D6 along with human CPR and cytochrome b_5_ (Baculosomes^®^) was the product of Thermo Fisher Scientific (Waltham, MA, USA).

### 2.3. Pooled Human Liver Microsomes

The pooled HLM preparation obtained from 10 donors (mixed gender) with a history of chronic alcohol exposure (lot FVT) was purchased from BioIVT Corporation (Baltimore, MD, USA) and referred to here as HLM (FVT). We also studied three preparations of pooled human liver microsomes from 50 donors (mixed gender) without a reported alcohol exposure history (the lots EGW, DNJ, and CDN). The sample referred to as HLM (Xen263) is the pulled preparation from 50 donors (mixed gender) supplied by XenoTech Corp. (Kansas City, KS, USA) (lot 2110263), which is also now a part of BioIVT Corp. The suppliers of the HLM preparations used in our studies, BioIvt Corporation and XenoTech Corp., have declared to adhere to the regulations of the Department of Health and Human Services for the protection of human subjects (45 CFR §46.116 and §46.117) and Good Clinical Practice (GLP) (ICH E6) in obtaining the samples of human tissues used for producing the preparations of human subcellular fractions available from these companies.

The concentrations of CPR in microsomal membranes, which were used to calculate apparent turnover numbers of the P450-dependent demethylation, were determined based on the rate of NADPH-dependent reduction of cytochrome c at 25 °C. The effective molar concentration of CPR was estimated using the turnover number of 3750 min^−1^ [15].

### 2.4. High-Throughput Assay of P450-Dependent Demethylation

The process of developing this procedure is outlined in Section 3.1 under Results. The final protocol of the high-throughput assay with the use of an OT-2 liquid handling robot (Opentrons Inc., Brooklyn, NY, USA) and a Cary Eclipse fluorometer equipped with a plate reader accessory (Agilent Technologies, Santa Clara, CA, USA) is described below.

The ketamine metabolism experiments were conducted with a series of 12 ketamine stock solutions in the Incubation Buffer (0.1 M Hepes buffer pH 7.4 containing 60 mM KCl), with concentrations ranging from 0 to 2.8 mM prepared with serial dilution. This series of stock solutions provided the ketamine concentrations in the incubation mixture, decreasing from 700 to 0 µM with a dilution factor of 1.73333 (≈√3). The 2 mL plastic tubes with these solutions were placed in a 20-tube aluminum rack (20 × 1.5 mL tube rotor, Benchmark Scientific, Sayreville, NJ, USA) positioned in location 8 of the OT-2 deck. Location 9 contained a 96-Well Deep Well Plate with 1 mL V-Bottom wells (Azotta Corp., Claymont, DE, USA). The first row of this plate was filled with 500 µL of NADPH-generating system in each of the 8 wells. The NADPH-generating system contained 200 µM NADP, 2 mM glucose-6-phosphate, and 1 unit/mL of glucose-6-phosphate dehydrogenase in the Incubation Buffer. An empty 96-well plate (UltraCruz V-bottom black plate, Santa Cruz Biotechnology, Santa Cruz, CA, USA) was placed at position 7 of the OT2 deck. Positions 10 and 11 contained the racks with 300 µL and 20 µL pipet tips, respectively. The protocol of the OT-2 run starts with filling all wells of the incubation plate with 40 µL of the NADPH system using an 8-channel, 300 µL pipette. It is followed by adding 20 µL of ketamine solutions to the wells using a single-channel 20 µL pipette. The protocol of the OT-2 run was designed in such a way that the concentrations of ketamine in the wells on the plate were shuffled so that the order of the rows containing increasing concentration was 6 (0 µM ketamine), 12, 5, 11, 4, 10, 3, 9, 2, 8, 1, and 7 (700 µM in the incubation mixture). The OT-2-compatible protocol file is available from the authors on request.

The plate prepared as described above was placed into a heater-shaker module equipped with a PCR-plate adapter and placed in location 1 of the deck. Suspensions of microsomes were diluted to the desired concentration (0.1–0.5 µM P450 for recombinant enzymes and 1–2 mg/mL for HLM samples) and placed into all eight wells of the first row of a PCR plate (96-well semi-skirted PCR plate, BrandTech Scientific Inc., Essex, CT, USA), 255 µL per well. The PCR plate was inserted into a pre-cooled PCR-plate cooling block (Eppendorf, Hamburg, Germany) and placed at position 5 of the OT-2 deck. An Azzota deep-well plate placed in location 6 contained 0.4 mL of quenching solution in each well of the first row. The quenching solution comprises 0.25 M acetoacetanilide and 1.5% acetic acid in ethanol. The second row of the same plate contained 0.6 mL of a 7 M solution of ammonium acetate per well. A rack with 300 µL pipette tips was inserted into deck position 4. The protocol of the OT-2 run of the activity assay starts with pre-incubation of the reaction plate in the heater-shaker module set at 1200 rpm shaking and 37 °C. This setting of the heater resulted in a temperature of 29.5–31 °C in the incubation mixture stabilizing after 10 min of pre-incubation. Subsequently, the robot starts the reaction by dispensing 20 µL of microsomal suspension per well with an 8-channel, 300 µL pipet. The addition of microsomes to each row was followed by 20 s of shaking. After adding microsomes to the last row, the robot continues shaking at a constant temperature until reaching the desired incubation time (12 min). The reaction was then stopped by adding the quenching solution in a volume of 32 µL per well with an 8-channel, 300 µL pipette. The addition of the reagent to each row was followed by shaking for 20 s. The protocol then ends with adding 48 µL of the ammonium acetate solution per well with an 8-channel, 300 µL pipette. The OT-2-compatible protocol file for this procedure is available from the authors on request.

The incubation plate treated as described above was then centrifuged at 3800 rpm in a Beckman Allegra 6R centrifuge with a GH-3.8 swing-out rotor and multiwell-plate adapters. After 20 min of centrifugation, the plate was scanned with a Cary Eclipse fluorometer with a well-plate accessory. We acquired the spectra of fluorescence excitation in the 300–440 nm region with emission at 468 nm and excitation and emission slits set at 10 and 20 nm, respectively. The fluorescence remains stable for approximately 40 min.

### 2.5. Methods of Data Analysis

All preparatory manipulations with the series of fluorescence spectra, their analysis, and the subsequent fitting of the titration traces were performed using our custom SpectraLab software [16] (version 3.1.1). The latest version of the software package is freely available on the author’s website [17].

The series of the excitation spectra were first sorted in the order of the increasing ketamine concentrations, smoothed with a 15-point window 3rd-order polynomial smoothing, and resampled to the 300–430 nm region with a 2 nm step. The amplitude of the spectra was corrected to account for differences in the sensitivity of the plate reader between different wells of the 96-well plate. For this correction, we used a set of 96 correction factors obtained with a plate with all wells filled with 160 µL of a standard fluorescent dye solution (we used Coumarin-460 laser dye with excitation at 395 nm and emission at 468 nm). 

The corrected set of fluorescence spectra was then subjected to a principal component analysis (PCA) combined with the approximation of the first three principal vectors with a combination of standard excitation spectra of 1 µM 3,5-di-N-phenylacetyl-1,4-dihydrolutidine (DPDL, the product of Hantzch reaction with acetoacetanilide), 1 µM NADPH, and a prototypical spectrum of excitation of internal fluorescence of microsomes obtained with rat liver microsomes in the presence of AAA and acetic acid but in the absence of ammonium acetate. The set of these fluorescence standards is available from the authors on request. Our software then used the partitioning coefficients obtained from these approximations to resolve the fluorescence intensities of DPDL and NADPH in the wells. Since the concentration of NADPH in the wells was kept constant with the use of the NADPH-generating system, its fluorescence was used as an internal standard to correct for minor variations in the sensitivity of the plate reader due to possible differences in the level of the liquid between the wells (see Section 3.1.2 for more details). After normalization, the set was subjected to PCA, and the approximations of the first two principal components with our set of fluorescence standards were used to determine the FA in the wells. The SpectraLab scripts for automatized data pre-treatment and analysis are available from the authors on request.

### 2.6. S-Ketamine Demethylation Assays with LC-MS/MS Technique

Preparation of the well plates and incubation were carried out using an OT-2 robot following protocols similar to those described for FA detection. However, in this case, the AAA reagent in the quenching solution was replaced with 17.5 µM midazolam used as the internal standard. The addition of ammonium acetate was omitted. 

The quantification of norketamine formed in the *N*-demethylation of S-ketamine was performed using a Xevo-TQ-XS MS (Waters, Milford, MA, USA) coupled with a microflow M-Class UPLC. An Acquity UPLC HSS T3 column (100 Å, 1.8 μm, 1 mm × 100 mm) was used for metabolite elution. The mass spectrometer was operated in multiple reaction monitoring mode using an electrospray ionization source. The LC parameters were set at a 50 μL/min flow rate with a 1 μL injection volume and a column temperature of 40 °C. Mobile phases A (water with 0.1% formic acid) and B (acetonitrile with 0.1% formic acid) were used. The following gradient was applied: 70:30 from 0.0 to 0.9 min, shifting to 10:90 from 0.9 to 3.0 min, held at 10:90 from 3.0 to 5.5 min, shifting to 70:30 from 5.5 to 5.8 min, and remaining at 70:30 from 5.8 to 7.5 min. The mass spectrometer was set to the positive ion (ESI+) mode with a cone voltage of 35 V. The MRM transitions for norketamine and midazolam were *m*/*z* 224.1→207.1, 179.1, and 125.1 (collision energy (CE), 15 eV) and *m*/*z* 326.1→309.1, 291.1, and 244 (CE, 20 eV), respectively. For norketamine, an average of all three product ions was taken during data processing, whereas for midazolam, *m*/*z* 291.1 and 244 were chosen as the quantifier ions, and *m*/*z* 309.1 was used as a qualifier ion. Elution times for compounds were 2.4 min for norketamine and 4 min for midazolam. Quantification was accomplished using a linear regression fit to an external calibration curve prepared in tandem with the samples. Peak integration and quantification were performed using Skyline 22.2 (University of Washington). Calibration curves of norketamine and midazolam (final concentrations, 1–1000 nM) prepared in a mixture of water:acetonitrile (30:70 *v*/*v*) were used for calculating metabolite concentrations in HLM incubations.

## 3. Results and Discussion

### 3.1. Developing a Method for High-Throughput Assay of Cytochrome P450-Dependent Demethylation Reactions

#### 3.1.1. Selecting the Method for Formaldehyde Detection

**Figure 1:**
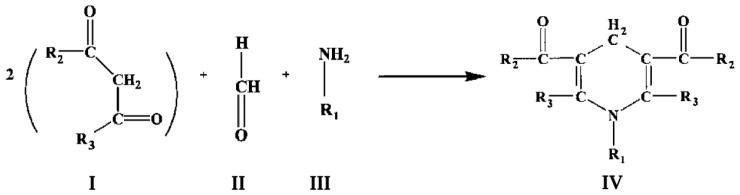
General scheme of the Hantzsch reaction with formaldehyde. In the case of the Nash method, R_1_, R_2,_ and R_3_ all remain for a hydrogen atom (H).

The most common method of detection of formaldehyde (FA) generated in P450-dependent demethylation reactions was developed in 1954 by Nash [6]. It is based on the Hanzsch reaction, which is illustrated in Figure 1. Here, two molecules of a β-diketone (I) react with a molecule of formaldehyde (II) and a molecule of ammonia or alkylamine (III) to form a molecule of a heterocyclic compound, a derivative of dihydrolutidine (IV), which absorbs in the near-UV or blue region and exhibits fluorescence with the maximum at 460–510 nm depending on the nature of substituents R_1_, R_2_, and R_3_:

The Nash method uses acetylacetone as β-diketone, which interacts with FA and ammonia, forming 3,5-diacetyl-1,4-dihydrolutidine (DDA). This product is detected by the appearance of absorbance at 412 nm. The reaction requires prolonged incubation at a high temperature (60 °C). In its original implementation, this method was the most frequently used technique for detecting formaldehyde formed in P450-dependent demethylation. This assay is very inexpensive and easy to perform. However, its sensitivity is limited by the relatively low extinction coefficient of DDA (7.7 mM^−1^cm^−1^ [6]). In practice, reliable quantification of FA with Nash reagent and absorbance measurements requires concentrations higher than 20 µM. This low sensitivity undermines the method’s utility in studies of P450-dependent reactions. Although detecting DDA fluorescence increases the method’s sensitivity [18], its fluorometric variant has been used only in one cytochrome P450-related study [19]. 

Another method of detecting formaldehyde that is sometimes used in P450 studies is a colorimetric determination of aldehydes with the purpald reagent (4-Amino-3-hydrazino-5-mercapto-1,2,4-triazole) [20,21]. However, in our trials, the sensitivity of this method was even lower than that of the Nash technique with fluorometric detection. 

Several kits for fluorometric determination of formaldehyde are also available from such manufacturers as Abcam, AAT Bioquest, and Arbor Assays. They are based on the use of undisclosed reagents and are quite expensive ($3–5 per assay). Most of them are not designed for use in P450 activity measurements. The only exception is the P450 Demethylation Fluorescent Activity Kit from Arbor Assays (product K011-F1). Unfortunately, the documentation provided by the manufacturer does not include exact information on its sensitivity. We probed using this kit in the assays in the presence of rat liver microsomes and found its sensitivity to be around 10 µM FA, which is only slightly better than that of the Nash method. This low sensitivity and the high price of the kit undermine its utility for P450 studies—we found only one published paper with its use [22].

Another commercial kit that we attempted to use is the Amplite^®^ Fluorimetric Formaldehyde Quantitation Kit from AAT Bioquest (catalog number 10057). We modified the protocol for use with microsomal preparations and tested the method using a conventional fluorometer with a 3 × 3 mm quartz cell. Although the sensitivity and reliability of this assay were better than those of the Arbor Assays kit, we held back from using it due to its high price.

There have also been several new fluorometric techniques for FA detection developed in the recent decade [10,23,24,25,26]. However, these new methods use custom reagents, which are not commercially available. They have never been used for P450 activity detection in the literature. To probe one of the most promising new methods, we synthesized the naphthalimide-based fluorescent probe RBNA, introduced by Jiang and co-workers [26]. Unfortunately, we found it extremely unstable and prone to spontaneous oxidation, preventing its practical use. 

In our further search for a reliable, low-cost FA detection method adaptable to high-throughput applications, we turned back to fluorometric variants of the techniques based on the Hantzsch reaction. Probing the classical Nash method with the detection of DDA fluorescence, we found that, when used with a conventional fluorometer, it gives a sensitivity of around 10 µM FA, which is only slightly better than its implementation with the absorbance detection. In addition, this method requires prolonged incubation of samples at 60 °C, which complicates its high-throughput implementation.

In addition to acetylacetone used in the Nash method, several other commercially available β-diketones were probed as reagents in fluorometric variants of the Hantzsch reaction [27]. According to Li et al. [27,28], the best sensitivity is obtained with methyl acetoacetate (MeAA) and acetoacetanilide (AAA). The respective lutidine derivatives have the highest extinction coefficients and fluorescence quantum yields. We first tried the variant with MeAA and found it much more sensitive than the original Nash procedure. The variant of this method with a conventional fluorometer and a 3 × 3 mm quartz cell allows reliable detection of 1 µM FA in the presence of microsomal preparations. However, similar to the Nash method, the Hantzsch reaction with MeAA requires prolonged incubation at 60 °C, which complicates its high-throughput implementation because of evaporation from the wells and condensation of the liquid on the well-plate cover. This effect may unevenly affect the volume of the liquid in the wells and thus undermine reproducibility.

In contrast, the reaction with AAA requires only 20 min of incubation at room temperature to be complete. Its sensitivity is even higher than that of the MeAA variant. Thus, we finally chose to use the AAA-variant of the Hantzsch reaction in further elaboration of the FA-detection method. It is to be noted that the same chemistry is apparently used in the FA-detection kit ab272524 available from Abcam and the formerly available kit ab133084 from the same manufacturer [29]. However, these kits are not optimized for enzyme activity assays with microsomes, and their high price precludes large-scale implementation in research.

#### 3.1.2. Elaborating a Method for Detecting Cytochrome-P450 Dependent Generation of Formaldehyde with the AAA Reagent

Our work on developing an AAA-based assay procedure was based on the publication of Li and co-workers [28]. According to these authors, the best sensitivity of the fluorometric assay with AAA, which is not readily soluble in water, is achieved in the presence of 30% ethanol. The optimal concentration of AAA recommended by Li et al. is 40 mM. In our optimization trials, we found that the ethanol concentration may be decreased to 20% without a loss of sensitivity, while the increase in the concentration of AAA to 50 mM decreases the incubation time needed. Following the recommendation of Li et al., we kept the concentration of ammonium acetate at 2.1 M.

Enzyme activity assays require the reaction to be quenched at the end of the incubation time. As a quenching method, we selected acidification with acetic acid, which can be added along with the AAA reagent in an ethanol solution. Since the optimal pH range for the Hantzch reaction is 7–7.8 [28], the concentration of acetic acid must be adjusted to decrease the pH below 4, where no P450 activity is observed, while allowing the pH to return to neutral after the addition of ammonium acetate. 

After optimization, we came to the following protocol: The incubation of microsomes with substrate and NADPH is performed in 80 µL of 0.1 M Hepes buffer, pH 7.4. The reaction is stopped by adding 32 µL of a 0.25 M solution of AAA in ethanol containing 1.5% acetic acid. That results in 0.43% acetic acid in the medium and decreases its pH to 3.9. The subsequent addition of 48 µL of 7 M ammonium acetate alkalizes the media and brings its pH to 6.9, which is close to optimal for the Hantzsch reaction. The reaction is complete after 20 min of incubation at room temperature. This time is used to spin out the microsomes by centrifugation at 3800 rpm in a Beckman Allegra 6R centrifuge with a GH-3.8 swing-out rotor. After 20 min of centrifugation, the fluorescence remains stable for approximately 40 min. 

**Figure 2:**
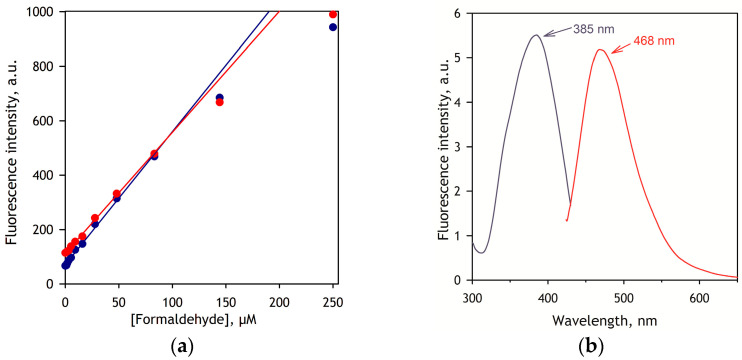
Detecting formaldehyde via Hantzsch reaction with AAA. (**a**) Calibration traces obtained in the absence (blue) and presence (red) of 0.5 mg/mL of rat liver microsomes. The fluorescence was measured at 468 nm (20 nm slit) with excitation at 395 nm (10 nm slit). The data points represent the averages of two experiments. Solid lines show linear approximations of the initial parts (0–83 µM) of the traces. (**b**) Spectra of excitation (blue) and emission (red) of the product of the Hantzsch reaction (DPDL) taken in the presence of 0.5 mg/mL of rat liver microsomes Excitation and emission wavelengths were set to 395 nm and 468 nm, respectively. Both spectra are normalized to 1 µM formaldehyde in the incubation media.

The calibration curves of the assay and the spectra of excitation and emission of DPDL are shown in Figure 2. As seen from this figure, the dependence of the intensity of fluorescence on the concentration of FA remains linear up to 85 µL FA, after which point the slope starts to decrease. 

As shown in Figure 2b, in the presence of microsomes, the product of the Hantzsch reaction, 3,5-di-N-phenylacetyl-1,4-dihydrolutidine (DPDL), exhibits the maxima of excitation and emission at 385 and 468 nm, respectively. However, since the assays of P450-dependent demethylation require the presence of NADPH, which is also fluorescent at 468 nm, the best results are obtained with excitation at 395 nm, where the excitation of the NADPH fluorescence is negligible.

#### 3.1.3. High-Throughput Implementation of the AAA Method

A high-throughput implementation of our assay requires the reaction to be carried out in multi-well plates, which are then scanned in a fluorescence plate reader. An important impediment in this setup is light scattering from the samples, which remains significant even after partial sedimentation of microsomes by centrifugation. The design of the plate readers, where the emission light is collected from the surface of the liquid, makes them much more sensitive to the scattering of excitation light than the conventional fluorometers, where the signal is collected through the wall of a rectangular glass cell. Furthermore, the amplitude of the registered signal and its sensitivity to light scattering depend largely on the level of liquid in the wells. Even an insignificant variation in the volume of liquid may result in a considerable change in the intensity of the registered signal and interference from light scattering. Because both the volume of liquid in the cell and light scattering by microsomal particles may differ between the wells, these effects cause considerable bias in the points and significantly decrease the accuracy of plate-reader-based assays. Our efforts in developing the high-throughput assay were directed towards minimizing these intervening effects.

To minimize light scattering from the pellet on the bottom of the wells, we used black well plates with V-bottom UltraCruz 96-well V-bottom Fluorescence Microplates from Santa Cruz Biotechnology, Inc. (Dallas, TX, USA). Probing the sensitivity of the registered signal to variations in the volume in the well, we found that a volume > 150 µL per well is required to achieve minimal variations in the registered signal upon changes in the volume in ±10% limits. Thus, the sample volume of 160 µL was chosen for our protocol.

However, even after this optimization and careful tuning of the optics of our plate reader (a Cary Eclipse fluorometer with a plate-reader accessory), the bias of the points in the assays carried out with registering fluorescence at one wavelength remained significant, especially at low concentrations of generated FA. To cope with these variations, we decided to register the excitation spectra of the samples instead of measuring fluorescence at one wavelength. Furthermore, we saw a need to introduce an internal fluorescent standard used for the normalization of the spectra to compensate for possible variations in the sensitivity of light detection between the wells. As our assays require the presence of NADPH, which is also fluorescent at 468 nm but differs from DPDL by the spectrum of excitation, we used the fluorescence of NADPH as a reference for normalizing the spectra while keeping the NADPH concentration constant with the use of a NADPH-generating system. 

In practice, the intensities of fluorescence of DPDL and NADPH in each well of the substrate titration series were resolved using the prototypical spectra of their excitation, as described in Materials and Methods. Then, the amplitudes of spectra in the series were normalized by the intensity of fluorescence of NADPH in each well divided by the averaged NADPH fluorescence in the series. This normalized series has been used to determine the concentration of formaldehyde by approximating the excitation spectra by a linear combination of the prototypical spectra of excitation of NADPH and DPDH. This procedure is described in more detail in Section 2.

To automate the assays, we used an Opentrons OT-2 liquid handling robot equipped with a heater-shaker module. We found that for optimal results, the reaction should be started by adding microsomal suspension (20 µL per well) to pre-heated well plates containing buffer solution with added substrate and NADPH-generating system (60 µL per well) and kept in the heater-shaker module. After incubation under shaking for a desired time, the reaction is stopped by an AAA-containing stop solution (see above) in a volume of 32 µL per well. It is followed by adding 48 µL of a 7 M ammonium acetate solution. All additions are made with a 300 µL, eight-channel automatic pipette. The plates were then centrifuged for 20 min at 3800 rpm and subjected to scanning the excitation spectra in the 300–440 nm region (emission at 468 nm) with a fluorescence plate reader. 

When developing the protocols for the OT-2 robot, we noticed that one of the sources of bias in titration traces is the tendency of the robot to acquire air bubbles in some tips as the pipette progresses across the well plate. It is especially noticeable when dispensing a viscous solution of ammonium acetate. This effect may cause an underdosing of liquid on a part of the plate. To make our assays less sensitive to these effects, we decided to “shuffle” the substrate concentrations in the wells so that the first and the second half of the well plate (rows 1–6 and 7–12) both contain the wells with the lowest and highest substrate concentrations. In practice, the concentrations of substrate increasing from 0 to maximal were placed in the rows taken in the following sequence: 6 (no substrate), 12, 5, 11, 4, 10, 3, 9, 2, 8, 1, and 7 (maximal concentration). As a result, possible underdosing in the second half of the plate results only in a saw-like bias instead of a systematic deviation of the points. This modification considerably increases the reproducibility of the assays.

### 3.2. Applying AAA-Based Technique to Study Ketamine Demethylation by Human Cytochrome P450 Species

#### 3.2.1. Setting up and Validating the AAA Method Applied to Ketamine Demethylation

**Figure 3:**
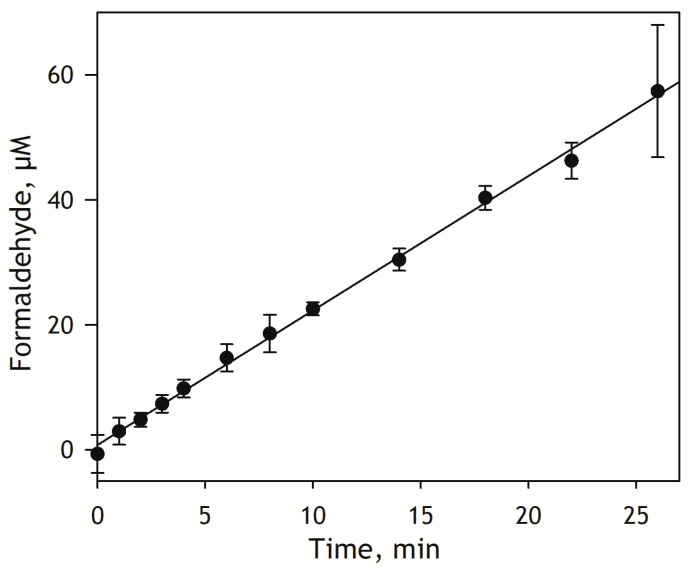
Kinetic trace of the accumulation of formaldehyde in the N-demethylation of S-ketamine by human liver microsomes. The experiment was performed with pooled HLM preparation (Lot 2110263) from XenoTech Corp. at 400 µM S-ketamine and 1.33 mg/mL of microsomal protein at 30 °C. The data points represent the average of four experiments, and the error bars show the respective standard deviations. The solid line corresponds to the linear approximation of the data set, which results in an estimate of the reaction rate of 2.15 ± 0.04 µM/min.

For any single-time-point kinetic assay, a critical step is selecting the incubation time to lay on the linear part of the kinetic trace of the product formation. To this end, we studied the kinetics of S-ketamine demethylation by human liver microsomes (HLM). According to Yanagihara et al. [30], the substrate saturation profiles of S-ketamine demethylation by HLM may be approximated with a combination of two Michaelis-Menten dependencies with *K*_M_ of 24 and 444 µM, respectively. Based on these estimates, for our kinetic assay, we selected a substrate concentration of 400 µM, which is close to the *K*_M_ of the low-affinity component. The kinetics of the generation of formaldehyde by pooled HLM preparation at 400 µM S-ketamine are shown in Figure 3. As seen from this figure, the kinetic trace keeps its linearity for at least 25 min. Based on this result, for our subsequent experiments, we select an incubation time of 12 min, which lies in the middle of the initial linear part.

To probe and validate our assay further, we compared the results of studying S-ketamine metabolism by recombinant CYP3A4 and CYP2B6 enzymes by two different methods: (i) the high-throughput FA-detection assay and (ii) the detection of norketamine with LC-MS/MS. In these experiments performed with the use of an OT-2 robot, we divided the incubation multi-well plates into two parts of four columns. In columns A-D, the quenching solution contained AAA. These wells were complemented with ammonium acetate and analyzed with the fluorescence plate reader as usual. In columns G-H, the AAA in the quenching solution was replaced with 17.5 µM midazolam, which we used as an internal standard. The addition of ammonium acetate was omitted for these columns, and the wells were analyzed with the LC-MS/MS technique (see Section 2).

**Figure 4:**
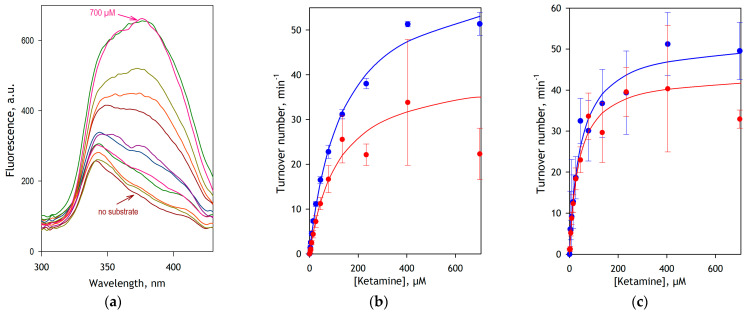
Demethylation of S-ketamine by recombinant CYP3A4 and CYP2B6 enzymes studied by fluorometric FA detection and LC-MS/MS. (**a**) A series of spectra of fluorescence excitation (emission at 468 nm) obtained in a fluorometric assay with CYP3A4-containing Supersomes^®^ (0.064 µM P450 in the incubation media) and S-ketamine concentrations increasing from 0 to 700 µM. (**b**,**c**) Substrate saturation profiles obtained with Supersomes^®^ containing CYP3A4 (**b**) and CYP2B6 (**c**) and S-ketamine with FA detection (blue) and LC-MS/MS (red) assays. The data points represent the averages of four replicates, and the error bars show the respective standard deviations. Solid lines represent the results of the approximation of the data sets by the Michaelis-Menten equation.

The results of these experiments are illustrated in Figure 4. In this figure, panel a exemplifies a series of fluorescence excitation spectra obtained in the assay with CYP3A4. As seen from this plot, these spectra feature a fairly invariant band of excitation of NADPH (λ_max_ = 340 nm) and an overlapping peak of excitation of DPDL (λ_max_ = 385 nm), which increases concomitantly with the increase in substrate concentration. The substrate saturation profiles obtained from the analysis of spectral series of this kind for CYP3A4 and CYP2B6 are shown in panels a and b in blue symbols. The data sets shown in red represent the results of LC-MS/MS assays. The results of fitting these traces to the Michaelis-Menten equation are compared in Table 1.

Comparing the data sets obtained by the two methods presented in Figure 4, it can be seen that the FA detection method is less prone to data scatter and provides better statistical reliability than the LC-MS/MS assay. The contrast between the two methods in their accuracy is particularly demonstrative in the case of CYP3A4 (Figure 4b).

As seen from Table 1, the *K*_M_ values for both CYP3A4 and CYP2B6 enzymes obtained by two different methods coincide up to the respective confidence intervals. At the same time, for both enzymes, the *V*_max_ values obtained from FA-detection assays are noticeably higher than those obtained from determining the formed norketamine by LC-MS/MS. This difference, which is especially significant in the case of CYP3A4, may reveal further conversion of norketamine to its hydroxylated metabolites by these enzymes. It should be noted, however, that while the ability of CYP2B6 to hydroxylate norketamine is already known [31], no metabolism of norketamine by CYP3A4 has been reported so far.

#### 3.2.2. Metabolism of Ketamine by Major Human Cytochrome P450 Species

To better elucidate the involvement of individual human P450 enzymes in ketamine metabolism, we used our novel high-throughput method to determine the kinetic parameters of S-ketamine demethylation by ten major cytochrome P450 species. In these studies, we used recombinant enzymes co-expressed with CPR and cytochrome *b*_5_ in the microsomes of insect cells. For all P450 species except CYP2D6, we used CYP-containing Supersomes^®^ from Gentest Corporation, while for the latter enzyme, we used Baculosomes^®^ Plus reagent from Thermo Fisher Scientific.

The results of these experiments are summarized in Table 2, where the P450 species are sorted in the order of decreasing intrinsic clearance (C_Lint_ = V_max_/K_M_) values. As seen from these data, in good agreement with the literature, the highest efficiency in ketamine demethylation is exhibited by CYP2B6. However, the *K*_M_ value for this enzyme obtained in our study is considerably higher than the estimates reported by Protmann et al. [31] (Table 2) and Wang et al. (10.2 ± 0.6 µM, [32]); it is more consistent with the estimate of 44.0 ± 0.6 µM obtained by Yanagihara et al. [30]. Our *V*_max_ value is also more consistent with that reported in the above study.

It has to be noted, however, that the comparison of the kinetic parameters obtained in recombinant systems, such as those provided in Refs. [30,31,32] and in the present study, is complicated by differences between the systems used by different authors. In our study, we used a commercial preparation of Supersomes^®^, similar to the study of Portman et al. [31]. In contrast, the study of Wang et al. [32] uses a custom baculovirus-based system, where the ratio of concentrations of cytochrome P450, its reductase, and cytochrome b5 (1:3.3:2.1) is quite different from that characteristic of commercial Supersomes^®^ (typically, 1:8:3, as estimated from the parameters of the individual lots of CYP2B6 Supersomes^®^ [33]). On the other hand, the study of Yanagihara et al. [30] uses microsomes from human B-lymphoblastoid cell lines, where the content of cytochrome P450 is 5–7 times lower than in Supersomes^®^. The differences between systems also explain a large bias in the estimates of kinetic parameters for CYP3A4 and CYP2B6 reported by different authors (Table 2). It should also be noted that the use of very long incubation times (60–180 min) without any control of the linearity of kinetic traces in the study of Portman et al. undermines the reliability of the estimates of *K*_M_ and *V*_max_ reported by these authors [31].

According to our analysis, the second efficient P450 species is CYP2C19, which has the highest affinity of all ten P450 isoforms studied here. This finding is notwithstanding the minor role in ketamine metabolism attributed to CYP2C19 by Yanagihara et al. [30] and Hijazi and Boulieu [34]. It is more consistent with the results of Portmann et al. [31], where the activity of CYP2C19 with ketamine was classified as the third after CYP2B6 and CYP3A4.

Two CYP3A enzymes occupy the next two places in our list of ketamine metabolizers. Although they exhibit quite similar parameters of S-ketamine demethylation, CYP3A4 is more efficient in this reaction due to its higher turnover number as compared to CYP3A5. Perhaps the most surprising finding is the relatively high efficiency of CYP2D6, which has the highest turnover number with S-ketamine of all the P450 species under study. It is notwithstanding the presumed minor role of this enzyme in ketamine metabolism [31,34]. *K*_M_ of 685 ± 124 µM, in combination with the high turnover number exhibited by CYP2D6, allows us to hypothesize that this enzyme is the main P450 isoform responsible for the low-affinity component of ketamine SSPs (*K*_M_ > 400 µM) observed in HLM [30,34]. Another unexpected observation is the low turnover number exhibited by CYP2C9. The low efficiency observed with this enzyme makes its presumed role in ketamine demethylation negligible, along with that of CYP1A2, CYP2C8, and CYP2E1.

It has to be noted, however, that the comparison of the kinetic parameters obtained in recombinant systems is insufficient for a steadfast attribution of the roles of the individual P450 enzymes in ketamine metabolism in HLM. It requires a comparative study with a large series of HLM preparations with the known composition of the P450 pool. These studies are now in progress in our laboratories.

#### 3.2.3. N-Demethylation of S-Ketamine by Human Liver Microsomes

**Figure 5:**
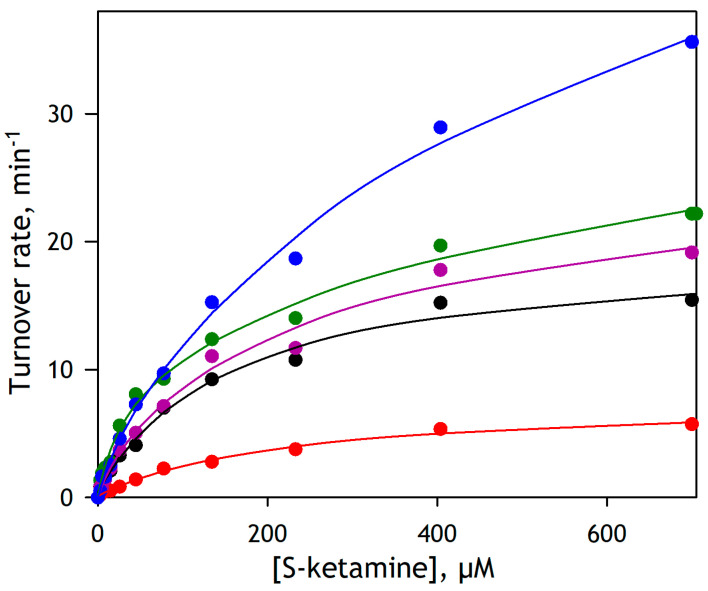
Substrate saturation profiles of N-demethylation of S-ketamine by five pooled preparations of HLM. The lots EGW (red), CDN (black), DNJ (magenta), and FVT (blue) were obtained from BioIVT Corp., and the 2110263 (Xen263, green) lot is from XenoTech Corp. The data points represent the average of four experiments, and the solid lines correspond to the approximations of the data sets with a combination of two Michaelis-Menten equations. The respective fitting parameters are shown in Table 3.

To probe the potential involvement of the individual P450 species in ketamine demethylation by the P450 ensemble of the human liver, we studied substrate saturation profiles of S-ketamine demethylation by five different pooled HLM preparations. The results of these experiments are illustrated in Figure 5. Of the five HLM lots exemplified in this figure, the lots EGW, CDN, and DNJ are the INVITROCYP 150-Donor (mixed gender) HLM obtained from BioIVT Corp. The lot FVT is a pooled preparation from 10 mixed-gender donors under chronic alcohol exposure obtained from the same supplier. Lot 2110263, designated hereafter as Xen263, is a pooled preparation from 50 donors (mixed gender) supplied by XenoTech Corp. The results of these experiments are illustrated in Figure 5 and Table 3. The rates of ketamine demethylation shown there are normalized on the concentration of CPR in HLM samples calculated from its activity in cytochrome c reduction (see Materials and methods) and thus expressed as mols of FA formed per minute per mole of CPR (min^−1^). 

The plots shown in Figure 5 reveal a contrasting difference between different HLM samples in both the amplitude and shape of the substrate saturation profiles of ketamine demethylation. Theoretically, these profiles may be considered a combination of multiple Michaelis–Menten dependencies corresponding to the individual P450 species involved in the reaction. However, resolving more than two Michaelis–Menten components from a single titration curve is barely possible. In this view, we fitted the titration curves shown in Figure 5 with a combination of two Michaelis–Menten equations to obtain a general idea of the possible involvement of individual P450s in ketamine metabolism. Parameters obtained from this fitting are shown in Table 3.

Table 3 shows that the HLM samples studied here may be divided into two groups. The SSPs of the preparations EGW and CDN that exhibit the lowest activity may be approximated with a single Michaelis–Menten equation with *K*_M_ values suggesting a predominate role of CYP3A enzymes with possible participation of some lower affinity enzymes, such as CYP1A2 or CYP2C9. In contrast, the SSPs of DNJ, Xen263, and FVT preparations reveal the participation of higher affinity enzymes, apparently CYP2B6 and CYP2C19, along with a high-amplitude low-affinity component, which is most likely associated with the involvement of CYP2D6. Notably, the FVT preparation obtained from the donors with chronic alcohol exposure has the highest activity with S-ketamine. It exhibits the highest fraction of the low-affinity component over all five probed HLM samples. 

## 4. Conclusions

This study introduces and describes a reliable, inexpensive, and versatile method for high-throughput kinetic assays of drug metabolism based on fluorometric quantification of formaldehyde (FA) formed in cytochrome P450-dependent demethylation reactions. We describe the high-throughput implementation of this technique applicable for automatized assays of cytochrome P450-dependent drug metabolism in human liver microsomes. The new method provides an efficient tool for large-scale screening of the drug metabolism in individual HLM samples from individual donors. The method may considerably facilitate and streamline routine studies of drug metabolism in the development of new pharmaceuticals. Furthermore, its combination with the toolset of proteomics offers a potent way for an in-depth analysis of correlations between the profile of drug metabolism and the composition of the drug-metabolizing ensemble, which is necessary for further elaboration of the concept of functional integration in human drug metabolism [2,35].

Application of our new method to studies of the metabolism of ketamine allowed us to shed new light on the involvement of multiple cytochrome P450 species in the oxidative demethylation of this anesthetic and antidepressant, which is increasingly used in the treatment of AWS and AUD. Probing the kinetic parameters of ketamine demethylation by ten major human P450s, we demonstrated that in addition to CYP2B6 and CYP3A enzymes, which were initially recognized as the primary metabolizers of ketamine, CYP2C19 and CYP2D6 also play an important role. At the same time, the involvement of CYP2C9 suggested in the previous reports appears insignificant. 

Studying the kinetics of ketamine demethylation by five pooled preparations of human liver microsomes, we observed dramatic differences in the kinetic parameters of this reaction between the HLM samples. Notably, the activity of the HLM preparation obtained from the donors with a history of chronic alcohol exposure was the highest of all HLM samples probed, and the shape of its substrate saturation profile (SSP) suggests an important difference from other HLM samples in the involvement of the individual P450 species. The apparent increase in the role of CYP2D6 and decrease in CYP3A involvement in ketamine metabolism will be further probed in a large-scale study combining a newly developed activity assay with the toolset of proteomics. These studies are currently underway. 

## Figures and Tables

**Table 1:** Parameters of S-ketamine metabolism by recombinant CYP3A4 and CYP2B6 obtained in parallel assays with fluorometric FA detection and LC-M/MS techniques *.

Parameter	CYP3A4		CYP2B6	
	FA Detection	LC-MS/MS	FA Detection	LC-MS/MS
*V*_MAX_, min^−1^	62.8 ± 2.2	40.6 ± 3.6	50.9 ± 2.9	43.9 ± 2.7
*K*_M_, µM	136.4 ± 13.2	107.3 ± 23.4	47.5 ± 10.6	36.9 ± 7.5

* The values in the table were obtained by fitting the averages of four individual datasets. The ± values represent the confidence intervals calculated for *p* = 0.05.

**Table 2:** Parameters of S-ketamine metabolism by recombinant P450 species determined in this study and their comparison with literature data *.

P450 Species	This Study			Yanagihara et al., 2001 [30]		Portmann et al., 2010 [31]	
	*K*_M_, µM	*V*_MAX_, min^−1^	CL_int_, µM^−1^min^−1^	K_M_, µM	V_MAX_, min^−1^	K_M_, µM	V_MAX_, min^−1^
CYP2B6	53.9 ± 14.4	50.6 ± 8.0	0.938	44.0 ± 9.6	33.0 ± 3.0	11.9	26.0
CYP2C19	19.3 ± 6.6	13.9 ± 7.1	0.719				
CYP3A4	113 ± 18	44.0 ± 7.7	0.390	399 ± 48	42.0 ± 14.0	61.2	39.0
CYP3A5	103 ± 22	27.5 ± 3.7	0.267				
CYP2D6	685 ± 124	68.0 ± 24.3	0.099				
CYP2A6	78.6 ± 29.9	5.6 ± 1.6	0.071				
CYP1A2	236 ± 48	9.5 ± 0.6	0.040				
CYP2C8	152 ± 49	5.6 ± 0.4	0.037				
CYP2C9	250 ± 76	6.2 ± 2.0	0.025	756 ± 85	43.0 ± 16.0		
CYP2E1	457 ± 182	5.3 ± 2.1	0.012				

* The values of the parameters obtained from our experiments shown in this table represent the averages of the results of 4–8 individual experiments. The ± values correspond to the confidence intervals calculated for *p* = 0.05. The Vmax values are expressed as mols of FA formed per minute per mole of cytochrome P450 (min^−1^).

**Table 3:** Parameters of S-ketamine metabolism by pooled preparations of human liver microsomes *.

HLM Lot Identifier	*K*_M1_, µM	*K*_M2_. µM	V_max_ (Total), min^−1^	Fraction of the Low-Affinity Component, %
EGW		225 ± 21	7.6 ± 0.3	100
CDN		159 ± 18	19.0 ± 0.9	100
DNJ	30.4 ± 14.0	298 ± 23	26.5 ± 1.3	86.3 ± 1.6
XEN263	31.5 ± 9.5	683 ± 84	35.8 ± 2.6	73.5 ± 7.0
FVT	33.0 ± 18.7	648 ± 40	64.9 ± 3.0	92 ± 1.2

* The values in the table were obtained by fitting the averages of four individual datasets. The ± values represent the confidence intervals calculated for *p* = 0.05. The Vmax values are normalized to the concentrations of CPR in HLM preparations and thus expressed as mols of FA formed per minute per mole of CPR (min^−1^).

## Data Availability

The data are contained within the article. The raw data sets used to generate the reported results are available from the authors upon reasonable request.

## References

[1] Mak K.K., Epemolu O., Pichika M.R. (2022). The role of DMPK science in improving pharmaceutical research and development efficiency. Drug Discov. Today.

[2] Davydov D.R., Prasad B. (2021). Assembling the P450 puzzle: On the sources of nonadditivity in drug metabolism. Trends Pharmacol. Sci..

[3] Guengerich F.P. (2001). Common and uncommon cytochrome P450 reactions related to metabolism and chemical toxicity. Chem. Res. Toxicol..

[4] Lewis D.F.V. (2003). Human cytochromes P450 associated with the phase 1 metabolism of drugs and other xenobioties: A compilation of substrates and inhibitors of the CYP1, CYP2 and CYP3 families. Curr. Med. Chem..

[5] Rendic S. (2002). Summary of information on human CYP enzymes: Human P450 metabolism data. Drug Metab. Rev..

[6] Nash T. (1953). The colorimetric estimation of formaldehyde by means of the Hantzsch reaction. Biochem. J..

[7] Heni N. (1971). Decrease of cytochrome p-450 after incubation with carbon tetrachloride in a NADPH regenerating system and partial conversion to cytochrome P-420. Experientia.

[8] Nebert D.W., Robinson J.R., Kon H. (1973). Further studies on genetically mediated differences in monooxygenase activities and spin state of cytochrome P-450 iron from rabbit rat and mouse liver. J. Biol. Chem..

[9] Hladová M., Martinka J., Rantuch P., Nečas A. (2019). Review of Spectrophotometric Methods for Determination of Formaldehyde. Res. Pap. Fac. Mater. Sci. Technol. Slovak Univ. Technol..

[10] Xu Z., Chen J., Hu L.-L., Tan Y., Liu S.-H., Yin J. (2017). Recent advances in formaldehyde-responsive fluorescent probes. Chin. Chem. Lett..

[11] Hurth K.P., Jaworski A., Thomas K.B., Kirsch W.B., Rudoni M.A., Wohlfarth K.M. (2020). The Reemergence of Ketamine for Treatment in Critically Ill Adults. Crit. Care Med..

[12] Garel N., McAnulty C., Greenway K.T., Lesperance P., Miron J.-P., Rej S., Richard-Devantoy S., Jutras-Aswad D. (2022). Efficacy of ketamine intervention to decrease alcohol use, cravings, and withdrawal symptoms in adults with problematic alcohol use or alcohol use disorder: A systematic review and comprehensive analysis of mechanism of actions. Drug Alcohol Depend..

[13] Long D., Long B., Koyfman A. (2017). The emergency medicine management of severe alcohol withdrawal. Am. J. Emerg. Med..

[14] Kryst J., Kawalec P., Pilc A. (2020). Efficacy and safety of intranasal esketamine for the treatment of major depressive disorder. Expert Opin. Pharmacother..

[15] Davydova N.Y., Dangi B., Maldonado M.A., Vavilov N.E., Zgoda V.G., Davydov D.R. (2019). Toward a systems approach to cytochrome P450 ensemble: Interactions of CYP2E1 with other P450 species and their impact on CYP1A2. Biochem. J..

[16] Davydov D.R., Deprez E., Hui Bon Hoa G., Knyushko T.V., Kuznetsova G.P., Koen Y.M., Archakov A.I. (1995). High-pressure-induced transitions in microsomal cytochrome P450 2B4 in solution—Evidence for conformational inhomogeneity in the oligomers. Arch. Biochem. Biophys..

[17] Davydov D.R. SpectraLab Software. http://cyp3a4.chem.wsu.edu/spectralab.html.

[18] Rapoport R., Hanukoglu I., Sklan D. (1994). A fluorimetric assay for hydrogen peroxide, suitable for NAD(P)H-dependent superoxide generating redox systems. Anal. Biochem..

[19] Yamaori S., Yamazaki H., Suzuki A., Yamada A., Tani H., Kamidate T., Fujita K., Kamataki T. (2003). Effects of cytochrome b(5) on drug oxidation activities of human cytochrome P450 (CYP) 3As: Similarity of CYP3A5 with CYP3A4 but not CYP3A7. Biochem. Pharmacol..

[20] Dickinson R.G., Jacobsen N.W. (1970). A New Sensitive and Specific Test for the Detection of Aldehydes: Formation of 6- Mercapto-3-substituted-s-triazolo [4,3-b]-tetrazines. J. Chem. Soc. D Chem. Commun..

[21] Li R.-J., Xu J.-H., Yin Y.-C., Wirth N., Ren J.-M., Zeng B.-B., Yu H.-L. (2016). Rapid probing of the reactivity of P450 monooxygenases from the CYP116B subfamily using a substrate-based method. New J. Chem..

[22] van Rensburg G.J., Bervoets L., Smit N.J., Wepener V., van Vuren J. (2020). Biomarker Responses in the Freshwater Shrimp Caridina nilotica as Indicators of Persistent Pollutant Exposure. Bull. Environ. Contam. Toxicol..

[23] Liu C., Cheng A.W., Xia X.K., Liu Y.F., He S.W., Guo X., Sun J.Y. (2016). Development of a facile and sensitive fluorimetric derivatization reagent for detecting formaldehyde. Anal. Methods.

[24] Wu Y., Zheng Z.M., Wen J., Li H.J., Sun S.G., Xu Y.Q. (2018). Imaging of formaldehyde in live cells and plants utilizing small molecular probes with large stokes shifts. Sens. Actuators B Chem..

[25] Dong B., Song X., Tang Y., Lin W. (2016). A rapid and facile fluorimetric method for detecting formaldehyde. Sens. Actuators B Chem..

[26] Jiang L.R., Hu Q., Chen T.H., Min D.Y., Yuan H.Q., Bao G.M. (2020). Highly sensitive and rapid responsive fluorescence probe for determination of formaldehyde in seafood and in vivo imaging application. Spectrochim. Acta Part A-Mol. Biomol. Spectrosc..

[27] Li Q., Sritharathikhum P., Oshima M., Motomizu S. (2008). Development of novel detection reagent for simple and sensitive determination of trace amounts of formaldehyde and its application to flow injection spectrophotometric analysis. Anal. Chim. Acta.

[28] Li Q., Sritharathikhun P., Motomizu S. (2007). Development of novel reagent for Hantzsch reaction for the determination of formaldehyde by spectrophotometry and fluorometry. Anal. Sci..

[29] Abcam ab133084—Formaldehyde Assay Kit. https://www.abcam.co.jp/ps/products/133/ab133084/documents/ab133084%20Formaldehyde%20Assay%20Kit%20protocol%20(web).pdf.

[30] Yanagihara Y., Kariya S., Ohtani M., Uchino K., Aoyama T., Yamamura Y., Iga T. (2001). Involvement of CYP2B6 in N-demethylation of ketamine in human liver microsomes. Drug Metab. Dispos..

[31] Portmann S., Kwan H.Y., Theurillat R., Schmitz A., Mevissen M., Thormann W. (2010). Enantioselective capillary electrophoresis for identification and characterization of human cytochrome P450 enzymes which metabolize ketamine and norketamine in vitro. J. Chromatogr. A.

[32] Wang P.F., Neiner A., Kharasch E.D. (2018). Stereoselective Ketamine Metabolism by Genetic Variants of Cytochrome P450 CYP2B6 and Cytochrome P450 Oxidoreductase. Anesthesiology.

[33] Discovery Life Sciences Gentest Resources. https://www.dls.com/biospecimens/admet/gentest-resources.

[34] Hijazi Y., Boulieu R. (2002). Contribution of CYP3A4, CYP2B6, and CYP2C9 isoforms to N-demethylation of ketamine in human liver microsomes. Drug Metab. Dispos..

[35] Dangi B., Davydova N.Y., Maldonado M.A., Ahire D., Prasad B., Davydov D.R. (2021). Probing functional interactions between cytochromes P450 with principal component analysis of substrate saturation profiles and targeted proteomics. Arch. Biochem. Biophys..

